# Information content best characterises the hemispheric selectivity of the inferior parietal lobe: a meta-analysis

**DOI:** 10.1038/s41598-020-72228-8

**Published:** 2020-09-15

**Authors:** Oliver Gray, Lewis Fry, Daniela Montaldi

**Affiliations:** Division of Neuroscience and Experimental Psychology - School of Biological Sciences, University of Manchester, Manchester, UK

**Keywords:** Neuroscience, Psychology

## Abstract

Our understanding of the inferior parietal lobe (IPL) remains challenged by inconsistencies between neuroimaging and neuropsychological perspectives. To date, others assume that hemispheric specialisation of the IPL is linked with the type of processing; attention processing in the right hemisphere; memory retrieval and semantic judgement in the left hemisphere. Here, we provide compelling evidence associating the type of information being processed with the recruitment of each hemisphere’s IPL. In a meta-analysis, we classify 121 previous fMRI reports of IPL activity arising from episodic memory retrieval, according to the type of information that characterises each fMRI contrast. We demonstrate that the left IPL is more consistently associated with retrieval of the semantic (95% of eligible contrasts) than perceptual aspects of memory (83%). In contrast, the right IPL is more consistently associated with the retrieval of perceptual (97%), than semantic aspects of memory (43%). This work revises assumptions of how the IPL contributes to healthy cognition and has major implications for IPL-related neuropsychological deficits.

## Introduction

Our understanding of the complex brain systems that support the encoding, storage, and retrieval of episodic memory, the what, where, and when of life’s events, has vastly improved with the advent of neuroimaging. However, for the last 15 years, the functional role played by the inferior parietal lobule (IPL), which includes the angular gyrus and supramarginal gyrus, in the retrieval of episodic memory has been the source of contentious debate^1–6^. Numerous functions, including the successful retrieval of episodic memories, have been strongly associated with activity in the IPL^7,8^. Despite the consistency of this association, an intriguing disconnect exists between the seemingly minimal effects of damage to the IPL on patients’ memory, and the IPL activity consistently observed during the retrieval of episodic memory in healthy participants^3,9,10^.

Memory researchers have offered several hypotheses to account for this theoretical discord between human lesion and neuroimaging observations^5,7,8,11–13^. Recently, Humphreys & Lambon Ralph^8^ considered these hypotheses and their supporting data together to generate the Parietal Unified Connectivity-biased Computation (PUCC) framework. Although PUCC provides interpretations of much of the data pertaining to parietal lobe functions, the framework, like most others that preceded it, provides limited insight into why neuroimaging studies frequently report parietal cortex activations that are lateralised to a single hemisphere of the brain. In this article, we consider previous models of parietal function and perform a meta-analysis to directly investigate this hemispheric lateralisation and its causes.

The attention to memory (AToM) model of parietal cortex function is one perspective that has divided scientific opinion, and both support and criticism has been provided by a mixture of investigative methods^4,11,14,15^. The AToM model proposes that the IPL performs a similar function in both spatial attention and memory retrieval^11,15^. Subregions within the IPL have been shown to regulate the reflexive allocation of attention to salient information^16,17^ and the AToM model proposes that the IPL supports bottom-up allocation of resources to the salient contents of medial temporal lobe output. More recent evidence has, however, identified spatial activation differences between IPL subregions that were separately associated with the allocation of attention and episodic memory retrieval^4,18,19^. These studies have shown that the AToM model is likely to be an overly simplistic explanation of IPL function. Building on the episodic buffer hypothesis^5^, the PUCC framework suggests instead that reflexive attention capture to sensory perceptions or episodic memories may emerge intrinsically as a function of the IPL integrating the spatial or temporal state of new perceptual or memory information with that of the current context^8^. Though more contemporary models explain parietal function more completely, the AToM model introduced the important idea that features of parietal function observed in one domain could be extrapolated to explain another^20^. This idea was central to the development of our working definitions of perceptually and semantically-defined memory experiences which we describe below.

It is well established that healthy individuals implicitly and preferentially allocate more spatial attention to the left visual field than the right; a phenomenon known as pseudoneglect (e.g.^21–25^). A large body of research attributes this effect to the specialisation of attention allocation processing by the right ventral attention network, including the IPL (e.g. ^16,21,26^). Pseudoneglect represents an example of a behavioural manifestation of hemispheric specialisation in these brain regions that might also be observed in memory. In contrast, the left hemisphere generally shows an obvious specialisation in language and semantic processing^27–29^. This specialisation has also been linked closely with the IPL with specific reference to the computation of word and sentence-level semantic information^30–32^. Here, we investigate the previous claim that memory retrieval processes also exhibit hemispheric specialisation of IPL function in the left hemisphere and propose a different specialisation based on the informational content of the representation being remembered.

We ask whether the hemispheric specialisations of the IPL, previously observed for spatial attention allocation^16,21,26^ and semantic processing^27,29–32^ are also evident for episodic memory processing. We performed a meta-analysis of previous fMRI investigations of episodic memory retrieval that observed IPL activations. These highly variable previous studies were classified according to whether they assessed the retrieval of either perceptual/experiential or semantic/conceptual aspects of episodic memories. As spatial attention allocation is intrinsically linked to the perceptual processing of incoming sensory information, predominantly in the visual domain, we hypothesised that the IPL shares a common hemispheric specialisation for retrieving detailed perceptual information from episodic memory and processing spatial attention. Similarly, we predicted that common hemispheric specialisations would be evident in the IPL during the retrieval of semantic or conceptual information from episodic memory and the processing of language. Accordingly, conditions in which performance depended on memory for the perceptual details of the original encounter, rather than semantic concepts, were expected to engage the right hemisphere IPL more than the left. Conversely, we expected left hemisphere IPL activations to be more prevalent than the right during semantic/conceptual retrieval of episodic memories.

Memory assessments were classified according to both the informational content of the memory that had been encoded and the specific requirements of the retrieval challenge at test. A perceptually defined memory experience was characterised by two primary features: 1) the encoding of detailed sensory information, most often in the visual domain, and 2) memory tests in which accurate performance was highly dependent on re-experiencing sensory information. For example, the detailed perceptual features of the encoded item (e.g. a semi-consumed glass of beer) are crucial to distinguishing between two similar variants of the same item (e.g. distinguishing between that glass of beer and a fuller one). Two very different criteria were used to classify a semantically/conceptually defined memory experience: (1) information stored in memory with limited perceptual detail, and (2) memory tests in which accurate performance could be achieved through memory for a semantic or conceptual label alone. For example, without needing to draw on the raw percept that led to that memory, one may accurately recognise that they had encountered the concept ‘beer’ earlier. To summarise, our classifications were based on the stimuli, encoding challenge, and retrieval challenge as well as the information represented in the fMRI contrast of interest (e.g., recall of visual features > narrative information would be classified as a perceptually defined memory experience).

## Methods—meta-analysis of IPL episodic retrieval memory effect

### Identification and eligibility

The process of identification, screening, and classification of eligible studies to create a representative sample of the literature is illustrated in the PRISMA 2009 flow diagram Supplementary Fig. 1. A brief summary of the encoding and retrieval methods, specific contrasts, and the resulting IPL activations for each study included in the review are provided in Supplementary Table 1 and 2.

**Figure 1:**
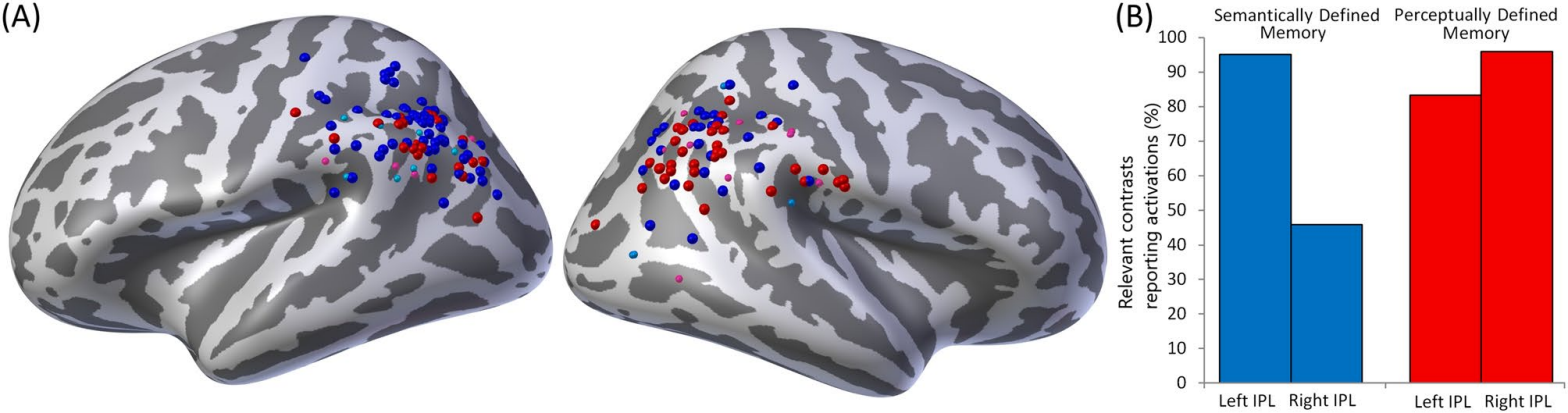
(**A**) IPL associated activations in semantically (blue), and perceptually (red) defined memory contrasts mapped onto an inflated template brain. Notes: Large spheres represent activations where the IPL associated activation was also the peak activation. Small spheres represent non-peak activations associated with the IPL. Readers should interpret the precise location of activations with caution as they reflect peak activations rather than illustrating the direction and extent of the activations across the brain. (**B**) A graphical representation of the percentage of IPL activations in the left and right hemisphere associated with semantically, and perceptually defined memory contrasts.

Studies published prior to October 2019 were identified through https://www.ncbi.nlm.nih.gov/pubmed searches. We targeted studies written in the English language for assessment of eligibility using the terms: memory, retrieval, fMRI and then checked that the abstracts of articles matching these keywords were episodic memory research. Where it was unclear in the abstract whether relevant data would be available, the full manuscript was assessed for eligibility. Studies included in the analysis contained at least one univariate episodic memory contrast with an activation specifically reported in the angular gyrus and/or supramarginal gyrus of the left and/or right hemisphere.

### Classification

A total of 65 eligible studies provided 121 contrasts for our analyses. We made a binary distintion (see our criteria above) between episodic memory contrasts that probed predominantly semantic/conceptual information at retrieval (76 contrasts) and those that required the retrieval of perceptual details (45 contrasts). Our criteria for classification were established and agreed collectively (OG and DM). A set of five studies (indicated in Supplementary Tables 1 and 1) were then used as a training set to improve the consistency of the classifications across the reviewers (OG, LF, DM). Two training studies were chosen randomly from each of the preliminary perceptual and semantic classifications of OG. A third study (Dobbins et al*.*^33^) was chosen deliberately to illustrate that our classifications should focus on fMRI contrasts rather than fMRI studies, and thus a single study could contribute both a perceptually and semantically defined contrast to the analysis. The classification of each contrast was then performed independently by each reviewer. Importantly, LF was blind to our hypotheses regarding the hemispheric specialisation of the IPL. After each reviewer had assessed and reported all contrasts, the reviewers met to discuss each contrast which was not agreed upon by all three reviewers (21 contrasts, OG-LF—16, DM-LF—14, OG-DM—9). After discussion and clarification of details of the tasks and contrasts reported in the respective papers, all these discrepancies were resolved.

We then further delineated the contrasts by considering the extent of the contribution of semantic and perceptual information to the memory retrieved. Each imaging contrast was rated 1 to 5, where 1 = definitively semantic retrieval, 2 = semantic retrieval with some perceptual retrieval, 3 = a near equal contribution of semantic and perceptual retrieval, 4 = perceptual retrieval with some semantic retrieval, 5 = definitively perceptual retrieval.

We also performed a further classification of perceptually and semantically defined retrieval contrasts, based on the contribution of recall/recollection or familiarity memory processing. This utilised the definitions and reports of these processes provided in the original studies. For this analysis, only contrasts where the memory retrieval episode was clearly more reliant on one type of memory (e.g. a remember > know paradigm) were included. Contrasts characterised by approximately matched levels of the same process (e.g. matched recollection—source memory for words that were seen > source memory for words that were heard) were excluded. We also excluded contrasts without a clear contribution of one of these processes over the other (e.g. recognition hits > correct rejections).

Lastly, we recorded the type of stimulus being remembered (visually presented words, images, other), the date of publication of each study, and we rated the stringency of the correction for multiple comparisons applied to each contrast of interest on a scale from 1–5: 1 = the most lenient corrections; 5 = the most stringent corrections. We differentiated between contrasts that utilised a lenient threshold (e.g. *p* < .05) and were uncorrected for multiple comparisons (1); contrasts with an uncorrected threshold of *p* < .001 and uncorrected cluster threshold (2); more stringent voxelwise and/or cluster extent thresholds without reference to false discovery rates (FDR) or family-wise error (FWE) corrections (3); voxelwise or cluster extent thresholds with FDR corrections (4); and those contrasts with family-wise error (FWE) corrected (voxelwise/cluster extent) thresholds (5).

### Analysis

The presence of IPL activation in each hemisphere was classified as the dependent variable. We assessed factors potentially affecting the probability of IPL activation using multiple regression analyses implemented in R^34^ using the glm package. Omnibus likelihood ratio tests and the subsequent assessment of specific model terms were conducted using the nagelkerke and Anova functions implemented in the rcompanion and car packages respectively^35,36^. Pseudo-R^2^ values were calculated using the nagelkerke function. These regression analyses assessed the probability of IPL activation in each hemisphere according to the contrast’s classification as semantically or perceptually-defined, the type of stimulus used, the year of publication of the associated study, and the stringency of correction for multiple comparisons. Assessment of the relationship between memory processes (e.g. recall) and IPL activations was excluded from the multiple regression analyses because of the limited availability of data in some conditions. Instead, summary comparisons of this data are provided in the results section. To ensure that our effects were not driven by multiple contrasts generated by one group of subjects from a single study, we generated bias-corrected and accelerated (BCa) bootstrap confidence intervals using the boot package^37^. This bootstrap approach used the strata function to repeatedly estimate the regression fit using a sample of one randomly selected contrast from each study. In Supplementary Table 1 and 2, we provide details of each contrast of interest, including the encoding and retrieval procedures that were performed by participants.

## Results

Imaging contrasts of memory paradigms that require retrieval of perceptual information show activation of the right IPL in 96% (43/45) of cases. The same contrasts elicit left IPL activations in 77% (35/45) of cases. Instead, during the retrieval of semantic or conceptual information, the left IPL is active far more consistently, 97% (74/76), than the same cortical area in the right hemisphere, 43% (33/76). Figure 1B illustrates the proportion of these semantically, and perceptually defined imaging contrasts that were associated with IPL activations in each hemisphere. Importantly, the stringency of corrections for multiple comparisons did not explain these differences. In the most stringently thresholded contrasts (ratings 4 & 5), paradigms requiring retrieval of perceptual information showed right IPL activation in 97% (34/35) of cases. Similarly, less stringently corrected contrasts (ratings 1 & 2) showed right IPL activiation in 80% (8/10) of cases. We identified left IPL activation in 82% (29/35) of stringent and 60% (6/10) of lenient perceptually-defined contrasts. Contrasts involving the retrieval of semantic or conceptual information displayed left IPL activation in 93% (31/33) of stringent and 100% of lenient cases. In contrast, the right IPL displayed activation in only 42% of stringent and 45% of lenient contrasts requiring semantic or conceptual information retrieval.

Two separate multiple logistic regression analyses assessed the probability of IPL activation in each hemisphere (one analysis per hemisphere) according to the contrast’s binary classification (semantic/perceptual), the stimulus type (visually presented words/images/other), the year of publication of the associated study, and the stringency of correction for multiple comparisons (1—least stringent to 5—most stringent). Both of these models both explained significantly more variance than a comparable null model (probability of left IPL activation—χ^2^(df H^0^-H^1^ = − 5) = 15.03, *p* = .01, pseudo-R^2^ = 0.24; probability of right IPL activation—χ^2^(− 5) = 22.19, *p* < .01, pseudo-R^2^ = 0.41). We observed significantly greater probability of activation in the left hemisphere’s IPL for semantically-defined than perceptual-defined memory retrieval contrasts (χ^2^(1) = 12.65, *p* < .01, mean estimate (*M*) = 2.96, bootstrap *M* = 4.28, CI [1.56, 19.95]). In contrast, probability of activation was significantly greater in the right hemisphere’s IPL for perceptually-defined than semantically-defined memory retrieval contrasts (χ^2^(1) = 38.77, *p* < .01, *M* = − 3.89, bootstrap *M* = − 5.01, CI [− 20.60, − 2.97]). Neither model observed a significant effect of stimulus type (left IPL—χ^2^(2) = 1.22, *p* = .54, average *M* = 0.60, bootstrap *M* = 0.80, CI [− 1.65, 0.61]; right IPL—χ^2^(2) = 4.60, *p* = .10, average *M* = − 0.47, average bootstrap *M* = − 1.81, CI [− 12.60, − 0.04]), the stringency of correction for multiple comparisons (left IPL—χ^2^(1) = 0.84, *p* = .35, *M* = − 0.42, bootstrap *M* = − 0.45, CI [− 0.82, − 0.02]; right IPL—χ^2^(1) = 1.68, *p* = .19, *M* = 0.28, bootstrap *M* = 0.30, CI [0.04, 0.60]), nor the year of publication (left IPL—(χ^2^(1) = 1.36, *p* = .24, *M* = 0.12, bootstrap *M* = 0.12, CI [0.03, 0.23]; right IPL—(χ^2^(1) = 0.50, *p* = .48, *M* = − 0.04, bootstrap *M* = − 0.04, CI [− 0.11, 0.02]).

The analyses presented in this section use our binary classification of episodic memory contrasts as either perceptually-defined or semantically-defined and assess the probability of activation in the IPL as a whole. In Supplementary Analysis 1, we provide the results of further logistic regression analyses that assessed activation probabilities according to our five point ratings (1 = definitively semantic, 3 = a near equal contribution, 5 = definitively perceptual) of the semantic and perceptual contributions to each memory retrieval contrast. In Supplementary Analysis 2, we describe the probability of SMG and ANG activation separately according to both the binary and rating based classifications. In these supplementary analyses, the factors affecting the probability of right IPL activation in the primary analyses were also associated with the probability of activation of both right IPL subregions. The more subtle and noisy effects in the left IPL (see estimates and χ^2^ values, reasons for this additional noise are explored in the discussion) were not observed in the left IPL subregions with the limited power available (average 40% decrease in activation frequency) for these analyses.

We further explored the extent of the hemispheric lateralisation of IPL function by assessing whether unilateral activations were more probable than bilateral activations for semantically- and perceptually-defined memory retrieval. Multiple regression models assessed this probability according to the same factors described in the main analyses (i.e., the contrast’s classification [semantic/perceptual, binary and 1–5 rating based classifications], the stimulus type, the year of publication, and the stringency of multiple comparison correction). Both types of information content classification produced models of unilateral left IPL activation probability that were better than a comparable null model (Binary—χ^2^(− 5) = 17.86, *p* < .01, pseudo-R^2^ = 0.37; Ratings—χ^2^(− 5) = 26.10, *p* < .01, pseudo-R^2^ = 0.28). In each of these models, semantically-defined memory retrieval contrasts were more likely than perceptually-defined contrasts to produce unilateral left IPL activations rather than bilateral activations (Binary—χ^2^(1) = 31.35, *p* < .01, *M* = 3.65; Ratings—χ^2^(1) = 21.73, *p* < .01, *M* = 1.20). Our assessment of the probability of unilateral right IPL activity revealed that only the model including our proportion-based classification of semantic/perceptual information content explained significantly more variance than a comparable null model (Binary—χ^2^(− 5) = 6.96, *p* > .05, pseudo-R^2^ = 0.15; Ratings—χ^2^(− 5) = 11.15, *p* = .048, pseudo-R^2^ = 0.23). In the proportion-based classification, right IPL activation was significantly more likely to be unilateral than bilateral with contrasts increasingly defined as more perceptual than semantic (χ^2^(1) = 9.91, *p* < .01, *M* = 1.11). These results provide strong evidence that rather than one type of information content being lateralised to one hemisphere and the other being associated with bilateral activations, semantic and perceptually-defined memory experiences are associated with left and right lateralised IPL activation respectively. Moreover it suggests that bilateral activations are produced by the presence of both types of information content in a memory experience. Full details of these analyses are presented in Supplementary Analysis 3.

The coordinates of all IPL activations associated with each contrast were recorded, and those contrasts that were given a clear semantic/conceptual classification (ratings 1 and 2, 64 contrasts) or a clear perceptual classification (ratings 4 and 5, 38 contrasts) are mapped onto an inflated template brain shown in Fig. 1A using the Multi-Modal Neuroimaging Analysis & Visualization Tool (MMVT^38^).

As well as classifying fMRI contrasts according to the dominant informational content for each contrast (perceptual or semantic), we also explored whether hemispheric specialisation in the IPL differed according to whether recollection and familiarity memory processing predominated. Direct comparisons are presented here because the limited availability of data in some classifications prevented the regression models from converging on reliable estimates, precluding the factor from inclusion in the analyses. With respect to semantic contrasts, we observed that recall or recollection-based retrieval produced left IPL activations in 96% (27 of 28) of eligible contrasts, whereas the right IPL was only active in 29% of these contrasts (8/28). Semantically defined familiarity memory contrasts displayed the same lateralisation as recollection; the left IPL displayed activation in 100% of these contrasts (12/12), compared with only 33% (4/12) showing right IPL activation. With respect to perceptual contrasts, 91% (20/22) of those involving recollection displayed right IPL activation, while left IPL activation was found in 77% (17/22) of these contrasts. Perceptually defined familiarity memory produced bilateral IPL activations in all 3 relevant contrasts (100%). However, this should be interpreted with great caution due to the small number of data points.

## Discussion

This review has identified a clear relationship between the hemispheric lateralisation of IPL activations and the informational nature of the target memory in fMRI investigations of episodic retrieval. Our findings suggest that the left IPL supports the retrieval of the semantic and conceptual aspects of episodic memory, whereas the right IPL supports in the retrieval of the perceptual features of a memory, as illustrated in Fig. 1A. Moreover, the information content of the memory provides a more accurate and complete account of IPL specialisation than the type of stimulus being remembered (e.g. words or images). Hemispheric specialisation in the IPL is well established in both the spatial attention^16,17,26^ and language processing^29,31,33^ literatures, and the lateralisation pattern we have observed here, whilst produced by different cognitive processes, is highly compatible with this previous work. Whilst clearly recognising the potential anatomic distinction in the IPL between the systems dedicated to different areas of cognitive function^39^, the information content interpretation links the brain mechanisms that support memory retrieval, with those that underpin spatial attention and language processes.

There are studies that offer a particularly clear insight into the hemispheric specialisation of memory retrieval function in the IPL. Some studies specifically focus on semantically-defined memory experiences and report unilateral left hemisphere IPL activations. For example, Frithsen and Miller^40^ presented participants with noun words and encouraged their encoding using a semantic judgement task. This study reported source recognition related activation in a highly left-hemisphere lateralised network of cortical regions that included the left IPL. Other studies focus specifically on perceptually-defined memory experiences and report unilateral right hemisphere IPL activations. For example, St-Laurent and colleagues^41^ contrasted perceptually rich video clips with minimal dialog with perceptually impoverished narrations of the same events, and Klostermann and colleagues^42^ tested the memory of participants using non-verbal, agrammatical music stimuli. Both studies reported activation of the right, but not left IPL. Interestingly, Dobbins & Wagner^33^ provided an interesting comparison of the episodic retrieval mechanisms associated with semantic and perceptual source memories. A recognition contrast in which subjects recollected items that were encoded with a semantic judgement revealed left hemisphere lateralised activations of the IPL and other nodes of the core recollection network. In contrast, recollection of perceptual features of the memory was associated with comparable areas exclusively in the right hemisphere. The lateralisations described here are typical of those reported in our classifications.

As might be expected, a large number of contrasts (67/121) showed some bilateral activation of the IPL. We observed strong evidence indicating that, compared with bilateral activations, unilateral IPL activation was more probable in the left hemisphere during semantically-defined memory retrieval and in the right hemisphere during perceptually-defined memory retrieval. We therefore propose that the large number of bilateral activations reflect the presence of both perceptual and semantic information in the retrieved episode; illustrating a characteristic richness of memory that is common to so many episodic memories but is rarely investigated. We analysed the relationship between this memory content and IPL activity from studies across a period of almost twenty years. Many, and particularly early, fMRI experiments have used word stimuli, probably because of the comparative ease with which they could be developed, manipulated, and presented. This accounts for the greater number of contrasts categorised as requiring the retrieval of semantic rather than perceptual information in our analysis (76 semantic contrasts, mean year of publication = 2010, SD = 6 years, compared with 45 perceptual contrasts, mean year of publication = 2013, SD = 5 years, see Supplementary Table 1 & 2). The targeted investigation of the effects of specific kinds of memory retrieval process, (e.g., familiarity), has developed more recently, and this has likely contributed to the surprisingly limited data points on perceptually-based familiarity.

The angular gyrus is both a subregion of the inferior parietal lobule and a node of the default mode network (DMN) and it exhibits activation with a huge variety of tasks. Interestingly, three functional subregions within the left angular gyrus have been identified^43^. Two of these left-sided subregions have been implicated in a DMN role, where their activity changed in all tasks relative to fixation (increase in dorsomedial angular gyrus activity, decrease in middle angular gyrus activity). However, in the ventrolateral angular gyrus, activity specifically changed with a semantic matching task. Interestingly, a more recent study exploring functional connectivity during episodic retrieval, revealed substantial connectivity between the left angular gyrus and other non-medial temporal cortex nodes of the DMN^44^. In contrast, the right angular gyrus exhibited strong retrieval-related functional connectivity with the medial temporal lobe. Consistent with the research highlighted in this discussion, our targeted review of IPL activations, strongly suggests that the right IPL performs a particularly important functional role in episodic retrieval that until now has lacked widespread appreciation.

Many recognition memory tasks require very little retrieval of the perceptual features of an encoding experience. For example, in a recognition memory paradigm, if we have conceptual memory that a glass of beer was encoded, then remembering precisely what the beer looked like is not critical to its recognition when the beer is presented alongside an apple. Instead, the retrieval of semantic concepts is often sufficient for accurate recognition memory. Therefore, in many of the studies that observe IPL retrieval effects, semantic/conceptual memory alone could support accurate recognition performance. For example, the traditional Yes/No memory task has not utilised similar lures at test as standard. As a result, the representations of encoded and new items are generally semantically very different and can be successfully distinguished by a simple semantic label for each encoded target. As the hippocampus plays a key role in supporting associative memory^45,46^, it is likely to support the accurate associative retrieval of semantic labels and concepts encountered in a specific encoding context without drawing critically on the IPL. This may explain why patients with IPL lesions do not appear amnesic when assessed with many traditional memory techniques.

Left hemisphere IPL activations associated with episodic memory retrieval are observed with greater frequency than right IPL activations. We suggest that this difference in frequency of lateralisation reflects the contents of episodic retrieval rather than the selective specialisation of the left IPL for memory retrieval. Additionally, the left IPL is associated with greater connectivity with the DMN than the right IPL^44^. As a result, DMN-related activity that is associated with a factor outside of experimental control, e.g. variability in arousal/task engagement, may elicit left but not right IPL activity and be misattributed as demonstrating specific differences in memory processing between the hemispheres. Unfortunately, the potentially inflated frequency of left IPL activity by DMN functions combined with a lack of focus on the role of the right IPL in memory retrieval has led many recent investigations of IPL memory function to focus solely on the left IPL^2,4,6,48–49^. This has occurred at the expense of a targeted exploration of the functional role of the right IPL, potentially obscuring the observation of key findings.

In contrast to those many memory paradigms that can be successfully completed through the retrieval of semantic or conceptual details of an episode, other memory tasks can only be completed through the retrieval of detailed perceptual information from memory. Recognition tasks that use a target and highly similar lures (e.g., four apples) have this requirement^50^. In such tasks, retrieving the label of an object (e.g., apple) does not help the participant to distinguish the target from other similar variants of the same object. Indeed evidence of right hemisphere IPL activations has been observed with this type of task^51,52^. Dennis et al*.*^52^, used related lures (items within a semantic category, e.g., different cats) and showed greater activity in the right angular gyrus for remember false alarms (recall of some, but not diagnostic, perceptual features) than non-remember false alarms (including guesses). In another study utilising similar lures, the right angular gyrus showed a large activation cluster that was greater for correct rejection (retrieval of perceptual features to reject/detect novelty) than false alarm responses^51^.

Interestingly, causal studies, utilising patients and neurostimulation techniques, have shown evidence of a functional role of the IPL in the retrieval of perceptual experiences^3,10,49,53,54^. In contrast to memory for semantic/conceptual labels, these studies required retrieval and integration of multiple perceptual features. For example, Davidson et al.^3^, observed that this kind of memory retrieval was impaired in patients with lateral parietal cortex lesions. More specifically, perceptual details of autobiographical recall were reduced despite preserved descriptions of non-specific semantic details. In addition, the patients produced fewer ‘remember’ responses during a remember/know recognition task. Simons et al*.*^55^, observed that during a recognition memory task, memory confidence but not accuracy, was reduced in patients with IPL damage. Although this was interpreted as an impairment of subjective memory in these patients, a reduction in their objective memory for the perceptual details of these recollections, although not tested, may also explain this effect. Of note, Russell et al*.*^56^ recently reported that individuals with right hemisphere IPL damage and without unilateral hemispatial neglect symptoms were specifically impaired in recalling their self-perspective at encoding. Contrastingly, these individuals could recall other information about the episode that could be summarised with a label^56^, potentially enabling functional compensation by the left hemisphere’s IPL. These studies all suggest that perceptual feature information is more susceptible than semantic/conceptual information to memory impairment when IPL function is compromised. The idea that memory retrieval is dynamically modulated by the engagement of the IPL was recently corroborated by examples of augmented memory retrieval abilities following up-regulation of left angular gyrus activity with TMS^57^. Critically, further work should utilise the upregulating TMS approach developed in the lab of Voss and colleagues^57,58^ with the right hemisphere’s angular gyrus and establish whether this modulates the balance of the retrieval of semantic and/or perceptual aspects of memory.

Additional causal support for the hemispheric specialisation of IPL processing being dependent on information content is provided by the effect of parietal lesions on different types of paired recall. Levy and colleagues^59^ observed that angular gyrus lesions in particular were associated with pronounced deficits in perceptually-defined memory recall. Moreover, patients with right hemisphere parietal damage and perceptually-defined memory impairments retained, or potentially even improved, their semantically-defined recall abilities (Fig. 8 of ^59^). The findings of the current meta-analysis, which are not affected by the confounding effects of encoding impairments caused by aphasia and unilateral hemispatial neglect (extremely common in the patient lesion studies of the parietal cortex), together with the data of Levy and colleagues, strongly support the idea of semantic compensation by the left hemisphere’s parietal cortex for deficits in perceptual retrieval.

The data presented here suggest that the retrieval of memory based on either recollection or familiarity, both exhibit the same pattern of IPL hemispheric specialisation driven by informational content. This evidence is difficult to reconcile with the “cortical binding of relational activity” (COBRA)^13^ model and the very similar Contextual Integration Model^60^ of IPL memory function. These models propose that multifaceted information arising from memory is integrated by the IPL during memory retrieval^6,13,53^. Whilst these hypotheses offer explanations of memory supported by recollection, they fail to effectively integrate the findings of previous research that has observed IPL activations associated with familiarity-based recognition^40,61–64^. In the case of familiarity, incoming information is compared to a stored representation and unlike recollection, does not involve the retrieval of additional information from the study context^45^. As a result, integration of memory components should not be required for familiarity memory. The hypothesis proposed by the episodic buffer model, and more recently adopted into the PUCC framework^8^, states that any output from the MTL (familiarity or recollection) is likely to be temporally or spatially incongruent with the current context and that the IPL works to adapt the information to fit this context. Therefore, it would be interesting if future work directly investigated the effect of manipulating perceptually and semantically defined information content on familiarity memory experiences. Finally, it will be important for future models and frameworks of IPL function to also ensure that activations caused by, and impairments in, recollection and familiarity for both semantically and perceptually-defined memory information are accounted for and explained.

## Conclusion

In conclusion, this review proposes a novel characterisation of the IPL processing involved in the retrieval of rich everyday episodic memories. For the first time, we provide compelling evidence that the IPL of the two hemispheres perform similar but functionally dissociable roles in the retrieval of a complete episodic memory. The left IPL supports the retrieval of the semantic and conceptual aspects of episodic memory, whereas the right IPL supports the retrieval of the perceptual features of the memory. We also provide evidence that this relationship is not driven by differences between types of retrieval process (i.e., recall/recollection or familiarity) or simply by the type of stimulus originally encoded (i.e., words or images) but instead, is driven specifically by mnemonic informational content.

Neuroimaging studies frequently report activation in the IPL across a variety of cognitive functions. Despite these numerous reports and the prevalence of IPL damage in patients with lesions (e.g. stroke) and disease (e.g. dementia), our understanding of its causal role has been limited by a lack of appreciation of those characteristics (e.g. hemispheric specialisation) which are common across cognitive functions. Future work should develop novel and more convergent approaches that utilise the methodological strengths derived from investigating these different cognitive domains. Moreover, it is crucial for a complete understanding of this area, that studies involving the direct modulation of brain activity (e.g. through TMS), target and compare effects of stimulation on *both* hemispheres. Only through this type of careful and strategic investigation, and the use of highly specified and controlled informational content, will we fully understand both the shared and selective roles of the IPL across different cognitive domains. Apart from informing a crucial area of neuroscientific knowledge, this work will be central to realising the enormous potential of therapeutically modulating IPL activity to improve function in the damaged and diseased brain.

## Supplementary information

Supplementary Information.

Supplementary Tables.
