# Supplementary material for: Information content best characterises the hemispheric selectivity of the inferior parietal lobe: a meta-analysis
Source: Sci Rep. 2020 Sep 15;10:15112. doi: 10.1038/s41598-020-72228-8 (PMC7493939; doi:10.1038/s41598-020-72228-8)
Supplement: Supplementary file 1 — Supplementary Information. [file 41598_2020_72228_MOESM1_ESM.docx]

Information Content Best Characterises the Hemispheric Selectivity of the Inferior Parietal Lobe: A Meta-Analysis

Oliver Gray*, Lewis Fry, Daniela Montaldi

Division of Neuroscience and Experimental Psychology - School of Biological Sciences

University of Manchester

Manchester

United Kingdom.

Correspondence should be addressed to O.G. ([oliver.gray@manchester.ac.uk](mailto:oliver.gray@manchester.ac.uk))

***
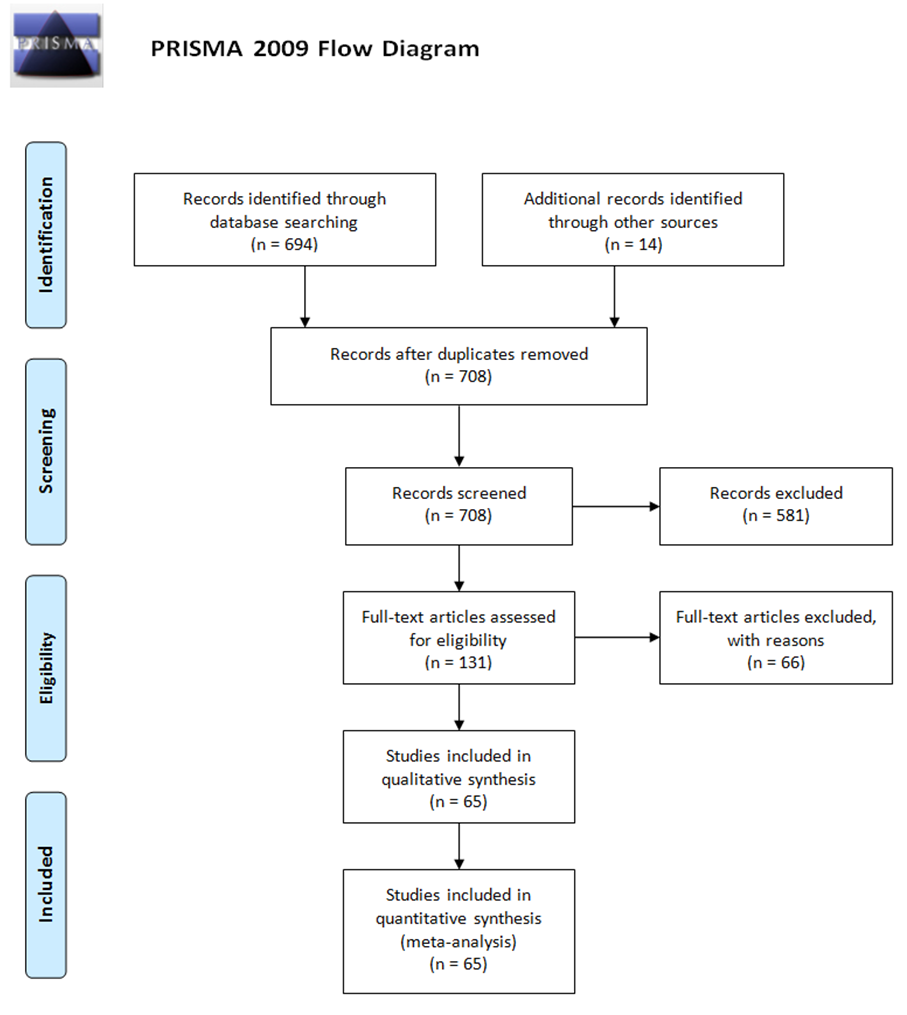
***

***Supplementary Figure 1:*** *A schematic representation of the process of identification, screening, and classification of eligible studiesfor the review of IPL activations associated with the perceptual and semantic aspects of episodic memory retrieval.*

**Supplementary Analysis 1 – Multiple Regressions including Proportion of Perceptually-/Semantically-Defined Memory Information**

Both models explained significantly more variance than a comparable null model (left IPL - χ^2^(df H^0^-H^1^ = -5) = 19.43, p < .01, pseudo-R^2^ = 0.31; right IPL – χ^2^(df H^0^-H^1^ = -5) = 35.31, p < .01, pseudo-R^2^ = 0.34). As in the binary analysis, left IPL activation probability was significantly higher for more semantically defined contrasts (χ^2^(1) = 17.05, p < .01, *M* = -1.31, bootstrap *M* = -1.49, CI = [-2.36,-0.74]). Neither stimulus type (χ^2^(1) = 3.08, p = .21, average *M* = -1.07, average bootstrap *M* = -1.07, CI = [-2.27, 0.32]), the stringency of correction for multiple comparisons (χ^2^(1) = 0.33, p = .56, *M* = -0.29, bootstrap *M* = -0.27, CI = [-0.76, 0.35]), nor the year of publication (χ^2^(1) = 1.80, p = .18, *M* = 0.14, bootstrap *M* = 0.16, CI = [0.05, 0.31]) significantly affected the probability of left IPL activation.

Again, as in the binary analysis, the probability of right IPL activation was significantly higher for more perceptually defined contrasts (χ^2^(1) = 29.6, p < .01, *M* = 1.34, bootstrap *M* = 1.40, CI = [0.94, 1.89]). In addition, stimulus type showed a significant association with the probability of right IPL activation (χ^2^(1) = 10.16, p < .01). This effect was driven by a significantly higher probability of right IPL activation for images than words (χ^2^(1) = 8.45, p = .01, *M* = 2.00, bootstrap *M* = 2.10, CI = [1.08, 3.13]). ‘Other’ stimulus types displayed a trend for higher probabilities of right IPL activation compared with words (χ^2^(1) = 4.40, p = .07, *M* = 2.08, bootstrap *M* = 2.17, CI = [0.59, 4.00]) but showed no evidence of a difference from images (χ^2^(1) = 0.01, p = .92 , *M* = 0.08, bootstrap *M* = 0.07, CI = [-1.15, 1.13]). Stringency of multiple comparison correction (χ^2^(1) = 2.52, p = .11, *M* = 0.34, bootstrap *M* = 0.36, CI = [0.09, 0.63]) and publication year (χ^2^(1) = 1.40, p = .24, *M* = -0.06, bootstrap *M* = -0.06, CI = [-0.13, -0.01]) were not associated with the probability of right IPL activation.

**Supplementary Analysis 2 – Multiple Regressions assessing the probability of SMG and ANG activation separately.**

*Left ANG – Binary Classification*

The model did not explain significantly more variance than a comparable null model - χ^2^(-5) = 7.00, p = .22, pseudo-R^2^ = 0.08. Further assessments were not made of this model.

*Right ANG – Binary Classification*

This model explained significantly more variance than a comparable null model - χ^2^(-5) = 15.41, p < .01, pseudo-R^2^ = 0.16. The right ANG displayed a significantly higher likelihood of activation in perceptually-defined than semantically-defined memory contrasts - χ^2^(1) = 10.80, p < .01. No other variables significantly affected the probability of right ANG activation. Stimulus type - χ^2^(2) = 0.15, p = .93; stringency of correction for multiple comparisons - χ^2^(1) = 1.19, p = .28; year of publication - χ^2^(1) = 0.98, p = .32.

*Left SMG – Binary Classification*

The model did not explain significantly more variance than a comparable null model - χ^2^(-5) = 10.37, p = .07, pseudo-R^2^ = 0.11. Further assessments were not made of this model.

*Right SMG – Binary Classification*

This model explained significantly more variance than a comparable null model - χ^2^(-5) = 19.70, p < .01, pseudo-R^2^ = 0.21. The right SMG displayed a significantly higher likelihood of activation in perceptually-defined than semantically-defined memory contrasts - χ^2^(1) = 14.60, p < .01. Stimulus type also significantly explained variance in the probability of activation in the right SMG - χ^2^(1) = 7.70, p = .02. No significant differences were observed between pictures, words, and other stimulus types in pairwise comparisons (pictures/words - χ^2^(1) = 4.38, p = .07; pictures/others - χ^2^(1) = 1.41, p = .24; words/others - χ^2^(1) = 5.67, p = .052). Stringency of correction for multiple comparisons and the year of publication did not significantly affected the probability of right ANG activation (stringency of correction - χ^2^(1) = 2.89, p = .09; year of publication - χ^2^(1) = 2.74, p = .10).

*Left ANG – Proportion Classification*

The model did not explain significantly more variance than a comparable null model - χ^2^(-5) = 8.66, p = .12, pseudo-R^2^ = 0.09. Further assessments were not made of this model.

*Right ANG – Proportion Classification*

This model explained significantly more variance than a comparable null model - χ^2^(-5) = 15.13, p < .01, pseudo-R^2^ = 0.16. The right AMG displayed a significantly higher likelihood of activation in perceptually-defined than semantically-defined memory contrasts - χ^2^(1) = 10.51, p < .01. No other variables significantly affected the probability of right ANG activation. Stimulus type - χ^2^(2) = 1.13, p = .23; stringency of correction for multiple comparisons - χ^2^(1) = 1.17, p = .28; year of publication - χ^2^(1) = 1.40, p = .24.

*Left SMG – Proportion Classification*

This model did not explain significantly more variance than a comparable null model - χ^2^(-5) = 10.83, p = .055, pseudo-R^2^ = 0.12. Further assessments were not made of this model.

*Right SMG – Proportion Classification*

This model explained significantly more variance than a comparable null model - χ^2^(-5) = 15.02, p = .01, pseudo-R^2^ = 0.17. The right SMG displayed a significantly higher likelihood of activation in perceptually-defined than semantically-defined memory contrasts - χ^2^(1) = 9.92, p < .01. Stimulus type also significantly explained variance in the probability of activation in the right SMG - χ^2^(1) = 7.34, p = .03. No significant differences were observed between pictures, words, and other stimulus types in pairwise comparisons (pictures/words - χ^2^(1) = 5.02, p = .07; pictures/others - χ^2^(1) = 0.67, p = .82; words/others - χ^2^(1) = 5.17, p = .07). Stringency of correction for multiple comparisons and the year of publication did not significantly affected the probability of right ANG activation (stringency of correction - χ^2^(1) = 2.94, p = .09; year of publication - χ^2^(1) = 3.26, p = .07).

**Supplementary Analysis 3 – Multiple Regressions assessing the probability of unilateral over bilateral IPL activations.**

*Left IPL – Binary Classification*

This model explained significantly more variance than a comparable null model - χ^2^(-5) = 17.86, p < .01, pseudo-R^2^ = 0.37. The subsequent ANOVA revealed that left IPL activation was significantly more likely to be unilateral than bilateral in contrasts defined as semantic than perceptual - χ^2^(1) = 31.35, p < .01, *M* = 3.65. The other factors included in the model were not significantly associated with the probability of a unilateral activation of the left IPL – stimulus type - χ^2^(2) = 3.42, p = .18; stringency of correction for multiple comparisons - χ^2^(1) = 1.21, p = .27; year of publication - χ^2^(1) = 0.58, p = .45.

*Left IPL – Proportion Classification*

This model explained significantly more variance than a comparable null model - χ^2^(-5) = 26.10, p < .01, pseudo-R^2^ = 0.28. The subsequent ANOVA revealed that left IPL activation was significantly more likely to be unilateral than bilateral with contrasts increasingly defined as more semantic than perceptual - χ^2^(1) = 21.73, p < .01, *M* = 1.20. We also observed a significant effect of stimulus type on the probability of unilateral activation - χ^2^(1) = 7.73, p = .02. Subsequent pairwise analysis of the three stimulus types revealed a significant difference between the probability of unilateral activation for contrasts associated with images and those associated with words - χ^2^(1) = 6.61, p = .03, *M* = 1.20. There was no significant difference between activation probabilities associated with the other stimulus types – image/other - χ^2^(1) = 0.01, p = .93; other/word- χ^2^(1) = 3.44, p = .13. The other factors included in the model were not significantly associated with the probability of a unilateral activation of the left IPL - stringency of correction for multiple comparisons - χ^2^(1) = 1.21, p = .27; year of publication - χ^2^(1) = 0.58, p = .45.

*Right IPL – Binary Classification*

This model did not explain significantly more variance than a comparable null model - χ^2^(-5) = 6.96, p = .22, pseudo-R^2^ = 0.15. Further assessments were not made of this model.

*Right IPL – Proportion Classification*

This model explained significantly more variance than a comparable null model - χ^2^(-5) = 11.15, p = .048, pseudo-R^2^ = 0.23. The subsequent ANOVA revealed that right IPL activation was significantly more likely to be unilateral than bilateral with contrasts increasingly defined as more perceptual than semantic - χ^2^(1) = 9.91, p < .01, *M* = 1.11. The other factors included in the model were not significantly associated with the probability of a unilateral activation of the left IPL – stimulus type - χ^2^(2) = 2.60, p = .27; stringency of correction for multiple comparisons - χ^2^(1) = 0.08, p = .78; year of publication - χ^2^(1) = 0.95, p = .33.
